# Hippocampal Transcriptome Changes After Subarachnoid Hemorrhage in Mice

**DOI:** 10.3389/fneur.2021.691631

**Published:** 2021-07-20

**Authors:** Angelique S. Regnier-Golanov, Friederike Dündar, Paul Zumbo, Doron Betel, Magda S. Hernandez, Leif E. Peterson, Eng H. Lo, Eugene V. Golanov, Gavin W. Britz

**Affiliations:** ^1^Laboratory of Cerebrovascular Research, Department of Neurosurgery, Houston Methodist Hospital, Houston, TX, United States; ^2^Applied Bioinformatics Core, Department of Physiology and Biophysics, Weill Cornell Medicine, New York, NY, United States; ^3^Applied Bioinformatics Core, Weill Cornell Medicine, New York, NY, United States; ^4^Division of Hematology and Medical Oncology, Department of Medicine, Weill Cornell Medicine, New York, NY, United States; ^5^Institute for Computational Biomedicine, Weill Cornell Medicine, New York, NY, United States; ^6^Center for Biostatistics, Houston Methodist Research Institute, Houston, TX, United States; ^7^Laboratory of Neuroprotection Research, Harvard Medical School, Massachusetts General Hospital, Charlestown, MA, United States

**Keywords:** subarachnoid hemorrhage, cognitive deficits, post-SAH syndrome, hippocampus, transcriptome, complement, oligodendrocyte

## Abstract

After subarachnoid hemorrhage (SAH), up to 95% of surviving patients suffer from post-SAH syndrome, which includes cognitive deficits with impaired memory, executive functions, and emotional disturbances. **Although these long-term cognitive deficits are thought to result from damage to temporomesial–hippocampal areas, the underlying mechanisms remain unknown**. To fill this gap in knowledge, we performed a systematic RNA sequencing screen of the hippocampus in a mouse model of SAH. SAH was induced by perforation of the circle of Willis in mice. Four days later, hippocampal RNA was obtained from SAH and control (sham perforation) mice. Next-generation RNA sequencing was used to determine differentially expressed genes in the whole bilateral hippocampi remote from the SAH bleeding site. Functional analyses and clustering tools were used to define molecular pathways. Differential gene expression analysis detected 642 upregulated and 398 downregulated genes (false discovery rate <0.10) in SAH compared to Control group. Functional analyses using IPA suite, Gene Ontology terms, REACTOME pathways, and MsigDB Hallmark gene set collections revealed suppression of oligodendrocytes/myelin related genes, and overexpression of genes related to complement system along with genes associated with innate and adaptive immunity, and extracellular matrix reorganization. Interferon regulatory factors, TGF-β1, and BMP were identified as major orchestrating elements in the hippocampal tissue response. The MEME-Suite identified binding motifs of Krüppel-like factors, zinc finger transcription factors, and interferon regulatory factors as overrepresented DNA promoter motifs. This study provides the first systematic gene and pathway database of the hippocampal response after SAH. Our findings suggest that damage of the entorhinal cortex by subarachnoid blood may remotely trigger specific hippocampal responses, which include suppression of oligodendrocyte function. Identification of these novel pathways may allow for development of new therapeutic approaches for post-SAH cognitive deficits.

## Introduction

Subarachnoid hemorrhage (SAH), blood accumulation in the subarachnoid space (1), in 85% of the cases results from aneurysmal rupture (2, 3). While SAH is the least frequent stroke disorder, accounting for 3–4% of all strokes (4), it has the most catastrophic outcome with 40% fatality (5). Fifty percent of cases occur in population under the age of 55, which otherwise has a good life expectancy (2, 6, 7). Ninety-five percent of survivors experience permanent disabilities and post-SAH syndrome, which includes depression, anxiety, impaired memory and executive functions, trouble of attention, and emotional and cognitive disturbances (5). Subsequently, a significant number, up to 45%, of SAH patients are unable to continue with their professional activities (7, 8).

Morphological brain damage following SAH includes atrophy of the temporomesial/hippocampal area (9, 10), which is associated with the long-term cognitive deficit and correlates with decreased neurocognitive scores (8). The role of hippocampal formation in cognitive processes and memory formation has been extensively documented (11). Following an aneurysmal rupture, most often localized at the base of the skull (12, 13), blood extravasates into the subarachnoid space damaging basal brain surface, including entorhinal cortices, which issue the main afferents to the hippocampus (14, 15). Lesions of entorhinal cortex lead to the damage of the perforant pathway and its hippocampal termination fields (16–18). We hypothesize that bleeding in the subarachnoid space triggers remote hippocampal damage, possibly by direct blood contact with the entorhinal cortex. Blood toxicity to the brain parenchyma has been well-established (19, 20). This would cause global changes in the gene expression resulting in the persistent disturbance of the hippocampal function.

We therefore used next generation RNA sequencing (RNA-seq) to explore specific changes in hippocampal transcriptome 4 days following SAH. Cellular and molecular perturbations accompanying early brain injury (within 72 h post-ictus) after sub-arachnoid have been well documented in the literature and reported non-specific response to brain insult (21, 22). However, mechanisms and respective pathways, underlying secondary brain injury, attributed to neurological and neurocognitive complications, taken place after 72 h have not been well characterized (23, 24). In the present study, we aim to explore biological processes and secondary mechanisms at the cusp between degeneration and regeneration happening at 4 days (25, 26) following SAH, closely mimicking delayed cerebral ischemia observed in humans (23) to explore processes at the origin of long-term consequences of SAH. Considering immense diversity of the various cells and their continuous interaction in the hippocampus, e.g., Iyer and Tole (27), this study was designed to gain an insight into leading biological processes occurring in hippocampus as a whole and to avoid changes in gene expression related to tissue processing for single-cell transcriptome analysis (28). Here, we aim to identify new therapeutic targets for the treatment of post-SAH syndrome.

## Materials and Methods

### Data Availability

The authors declare that all supporting data are available within this article and its Data Supplement. The RNA-seq data and the list of differentially expressed genes (DEGs) can be viewed at the Gene Expression Omnibus (GEO) under accession number GSE167110 (29). Scripts and code are uploaded to [Regnier-Golanov AS, (29)]. Other datasets generated for this study are available upon request to the corresponding author.

### Animal Procedures

Animal experimental procedures were conducted in accord with the U.S. National Institutes of Health “Guide for the care and use of laboratory animals” and in compliance with the ARRIVE guidelines. All experimental procedures were approved by the Institutional Animal Care and Use Committee of Houston Methodist Research Institute, Houston, TX (Protocol #: AUP-0220-0009). Animals were housed in the institutional animal facilities on a 12 h day/night cycle with *ad libitum* access to food and water.

### Unilateral SAH Induction

Studies were performed in 11 male C56BL/6J mice, 10–14 weeks old (Jackson Labs). To minimize the variability of the RNA-seq experiments, the present study was limited to the male animals. Animals from the same batch were randomly assigned to one of three groups: SAH (*n* = 4), Sham (*n* = 3), and Naïve (*n* = 3). Experimental SAH was induced by the unilateral perforation of the Circle of Willis as previously described (30). Anesthesia was induced with 3% isoflurane in air and maintained at 1.5–1.75% isoflurane in 80% N_2_/20% O_2_. For regional cerebral blood flow (rCBF) measurement, needle probe (1.5 mm diameter, Moor Instruments, Wilmington, DE) fixed to the stereotaxic holder was placed over the intact skull over the parietal cortex through the appropriately prepped (alcohol and chlorhexidine 2%) skin cut on the side of perforation, after animal anesthetization and placement in stereotaxic frame. Drop of mineral oil was applied to the skull for optical contact between the probe and the skull. Animal placed on a homeothermic blanket with the body temperature maintained at 37°C by the rectal probe feedback (Harvard apparatus) was rotated in a supine position. The heart rate was continuously monitored (LabChart, AD Instruments). Neck skin was shaved and prepped with alcohol and chlorhexidine (2%). In aseptic conditions through the midline neck incision, common, external, and internal carotid arteries were exposed. All three arteries were mobilized using silk thread (7–0). The external carotid artery was cut between two ligatures, and a stump was formed. To minimize the bleeding and avoid mortality, 6-0 prolene filament (length 12 mm) was advanced through the stump into the internal carotid and moved forward for ~0.5 mm beyond the point of slight resistance. The filament was withdrawn and stump ligated, and the wound was closed. In sham-operated mice, the filament was advanced to the resistance point and withdrawn without puncture (30). Wounds were closed, and animals were allowed to recover before returning to the home cage. Following the surgery, mice were monitored daily for 96 h. We experienced no procedural or post-surgery mortality when performing experiments in this study.

### SAH Severity and Neurological Assessment

Only SAH mice showing a rCBF drop by ≥50% (average 80 ± 15%, *P* < 0.001) immediately after perforation were selected for the study. A representative photograph of an SAH experimental animal done for the study is shown (Supplementary Figure 5).

Neurological assessment was done with the Garcia scale as previously reported (31) daily. No significant difference was observed in the baseline scores for the animals randomly assigned to either group [*H*_(3)_ = 3.6133, *P* = 0.1581, *n* = 3–4/group] at 24–96 h after surgery (Supplementary Table 2). Due to the limited number of animals, Garcia scale, initially developed for stroke neurological consequences evaluation (31), was not sufficiently sensitive to detect differences of neurological outcomes between Sham and SAH in our experiments, but to rather detect effects of surgery. Further neurobehavioral studies (*N* = 18 with *n* = 10 for SAH and *n* = 8 for Sham) in our laboratory detected no neurological impairments with the Garcia scale but instead showed significant neurocognitive deficits at 4 days after SAH with Y-maze, Social interaction, and Open-field test (in preparation).

### RNA Extraction and RNA Sequencing

Four days following surgery, mice were euthanized with CO_2_ followed by thoracotomy and decapitation. Immediately after bilateral whole hippocampus of SAH (*n* = 4), Sham (*n* = 3), and Naïve (*n* = 3) group was manually collected by an experimenter blinded to the specific group and total RNA was extracted immediately after using QIAzol reagent (RNeasy Mini Kit, Qiagen Inc., Hilden, Germany). RNA was quantified using the Nanodrop 1000 spectrophotometer (Thermo Fisher Scientific, Waltham, MA). RNA-seq was performed by RNA core of Weill Cornell Medicine (New York, NY). Total RNA integrity was evaluated with the Agilent Bioanalyzer 2100 (Agilent Biotechnologies, Santa Clara, CA), and only samples with an RNA integrity number (RIN) >9.0 were further processed for RNA-seq. All RNA-seq libraries were prepared using 100 ng of total RNA with the TruSeq Stranded Total RNA sample preparation kit (ribosomal RNA depletion and stranded RNA-Seq) with Ribo-Zero according to the manufacturer's specified protocol (Illumina, San Diego, CA). Samples were multiplexed and sequenced across multiple lanes of a HiSeq 4000 Sequencing System (Illumina) using 50-base paired-end reads to achieve a minimum depth of 30 million aligned read-pairs, which is sufficient to evaluate the similarity between transcriptional profiles according to ENCODE guidelines (32).

### RNA Sequencing Analysis

Before differential gene expression analysis, the quality of the sequences was assessed based on several metrics using FastQC and QoRTs (33, 34). All our samples passed the quality control (Supplementary Figures 1A–F). We aligned, with default parameters, the RNA-seq reads to the mouse reference genome (mm9) using STAR (35). Gene coverage estimates were obtained with featureCounts using composite gene models (union of the exons of all transcript isoforms per gene) from UCSC mm9 annotation from Illumina's iGenomes (36, 37). Uniquely mapped reads that unambiguously overlapped with no more than one Gencode composite gene model were counted for that gene model; the remaining reads were discarded. The counts for each gene model were used for the subsequent analyses.

#### Differentially Expressed Genes

DEGs were determined with DESeq2 (38), a statistical software package tailored to the gene-wise testing of expression differences using read count data. In brief, DESeq2 assumes an underlying negative binomial distribution of the read counts per sample and gene; it accounts for heteroskedasticity, differences in sequencing depth, and RNA repertoire, as well as for low sample numbers when estimating the expression differences between contrasts of interest.

#### Functional Analysis to Identify Gene Sets of Interest

Functional analysis to identify gene sets of interest was done using the compareCluster function of the clusterProfiler R package. The overrepresentation analyses (ORA) based on hypergeometric distribution with additional multiple hypothesis-testing correction (39, 40) were run with the gene sets of the Gene Ontology (GO) consortium (41) and REACTOME pathways (42). ORA was done for each group of significantly up- or downregulated DEGs. The results were represented as dotplots representing the *gene ratio* calculated *as the genes related to a given GO term/total number of DEGs*. This allowed us to visualize the most significantly overrepresented gene sets in our study. The top 20 enriched GO terms for each group were plotted. We also generated network-based representations of the most overrepresented gene sets using the cnetplot function of the clusterProfiler package, plotting the top five enriched gene sets. This allowed us to visualize which individual genes are shared or unique to individual gene sets.

#### Gene Set Enrichment Analysis

Gene set enrichment analysis (GSEA) was performed using the *fgsea* package against MSigDB Hallmark (Broad Institute) pathways (43). Genes were ranked by the DESeq2 Wald test statistic. Hallmark gene sets represent biological processes as defined by publicly available data sets such as microarrays and RNA-seq (44). A normalized enrichment score (NES) was calculated for each pathway, and the top pathways with FDR < 0.05 were displayed.

To identify putative master regulators orchestrating the expression of our DEGs expression, we used the Ingenuity Pathway Analysis suite (IPA; QIAGEN, Hilden, Germany). We used the upstream regulator analysis (URA) tool to identify the molecules whose activity may explain the observed gene expression changes (45). We mined for putative upstream regulators by filtering for genes with the highest activation *z*-score.

To test for the enrichment of DNA motifs in the promoters of DEGs, we used several tools. We used HOMER (v4.9.1) (46) with the flags-start −1,000 -end 50 to look only for motifs enriched within the promoters of genes (47) relative to all other promoters defined as −1,000 to +50 bp relative to each gene's transcription start site (TSS).

In the MEME suite (48), which offers several tools for DNA motif analyses, we extracted the DNA sequences representing 500 bp upstream of each TSS of both up- and downregulated DEGs. We first ran DREME, which discovers short, ungapped motifs that are relatively enriched in the sequences of interest compared to control sequences (47). DREME was run with default settings, using 500 randomly sampled promoter regions of genes that showed no significant change as control regions.

We then ran CentriMo (49), which identifies known or user-provided motifs that show a significant preference for particular locations. CentriMo takes a set of motifs and a set of equal-length sequences and plots the positional distribution of the best match of each motif (50). We used 500 randomly sampled promoter regions of genes that showed no significant change as control regions.

Finally, we used AME (51), which identifies user-provided motifs that are relatively enriched in the sequences of interest compared with control sequences (51, 52). In contrast to CentriMo, the location of the motif within the supplied set of promoter sequences is not considered. We ran AME *via* the web server using the promoter regions of the upregulated DEGs and to determine if known motifs were found to be enriched compared to promoter regions of genes without changes.

### Rt-qPCR for RNA-Seq Validation

Hippocampal tissue and RNA extraction were obtained identically to RNA-seq experiments. Male C56BL/6J mice, 10–14 weeks old (*N* = 18 with *n* = 9 for SAH, *n* = 9 for Sham), were used for this set of experiments. A preamplification step was simultaneously done with the reversed transcription (RT) step using the iScript Explore One-step kit (Biorad, Hercules, CA). Briefly, 1 μg of DNase-treated total RNA was reverse transcribed for 60 min at 45°C, RT was inactivated by increasing temperature to 95°C for 3 min, and 14 preamplification steps were done with the following thermocycler protocol: 95°C for 15 s and 58°C for 4 min, hold at 4°C. Primers (Supplementary Table 1) (Sigma-Aldrich, St. Louis, MO), designed with NCBI tool (53), and 2.5 μl of a custom preamplification assay pool of the reverse and forward primers, diluted at 200 μM, were added to the reaction. The preamplification samples were diluted 10-fold in TE buffer (Invitrogen, Carlsbad, CA). Quantitative PCR was run using 5 μl of diluted cDNA and SYBR green chemistry with the SsoAdvanced Universal SYBR green supermix on a CFX Connect thermocycler (Biorad, Hercules, CA) and a cycling program of 30 s at 95°C, and 40 cycles of 95°C for 15 s and 60°C for 30 s. Primers were used at a 300 nM final concentration in a 20 μl total volume. After completion of qPCR, a melting curve of amplified products was determined. Pgk1 was selected as our reference gene after comparing several common housekeeping genes (54) and our RNA-seq list of genes showing no difference in expression between the control and SAH group. In short, the mean of the threshold cycle (Ct) of three technical replicates was used as the Ct for the target of a given animal Δ*Ct*(*Animal*) = *Ct*(*Target*)–*Ct*(Pgk1), and fold changes were calculated using the following equation: 2^−ΔΔCt^ = 2^−[*MeanΔCt*(*SAH*)−*MeanΔCt*(*Sham*)]^ (55).

### Western Blot

Male C56BL/6J mice, 10–14 weeks old (*N* = 7 with *n* = 3 for SAH, *n* = 4 for Sham), were used for this set of experiments. Mouse bilateral whole hippocampus was homogenized in ice-cold RIPA buffer [25 mM Tris-HCl pH 7.6, 150 mM NaCl, 1% NP-40, 0.5% sodium deoxycholate, 0.1% SDS, 1X HALT Protease and Phosphatase Inhibitor Cocktail Solutions (ThermoFisher Scientific)], centrifuged, and stored at −80°C until use. Proteins were quantified by Pierce BCA Protein Assay Kit (ThermoFisher Scientific) and resolved by high resolution Nupage 4–12% Bis-Tris Gel (Thermo Fisher Scientific-Invitrogen). Anti-rabbit MOG (1:1,000; #12690-1-AP, Proteintech) was incubated overnight and diluted in blocking buffer (5% dried milk in Tris-buffered saline, pH 7.4, and 0.2% Tween-20, TBST). After no-stripping of the membrane, anti-rabbit GAPDH linked to HRP (1:2,000; #8884, Cell Signaling Technology) was incubated on the same membrane following MOG labeling overnight and diluted in blocking buffer. Detection was done with Pierce ECL detection reagent (ThermoFisher Scientific). Protein bands were detected with X-ray film (GenHunter.com), and densitometry was calculated after scanning the films at 600 dpi using ImageJ software and the Miller's tutorial (56). Results were expressed as percentage of change from Sham.

### Statistical Analysis

Graphs were plotted and statistical analysis was done with GraphPad Prism 9 (GraphPad Software, San Diego, CA). Shapiro–Wilk test was used for test of normality, and *F*-test for heterogeneity of the variance. Parametric data are represented as mean ± SD, and nonparametric as median with interquartile range. Statistical significance was set at *P* < 0.05. Pairwise comparisons were analyzed using a two-tailed *t*-test. For the Garcia scale, a Kruskal–Wallis test was done. Statistics are reported in the text and figure legend.

## Results

### Changes in Differentially Expressed Genes

**Figure 1:**
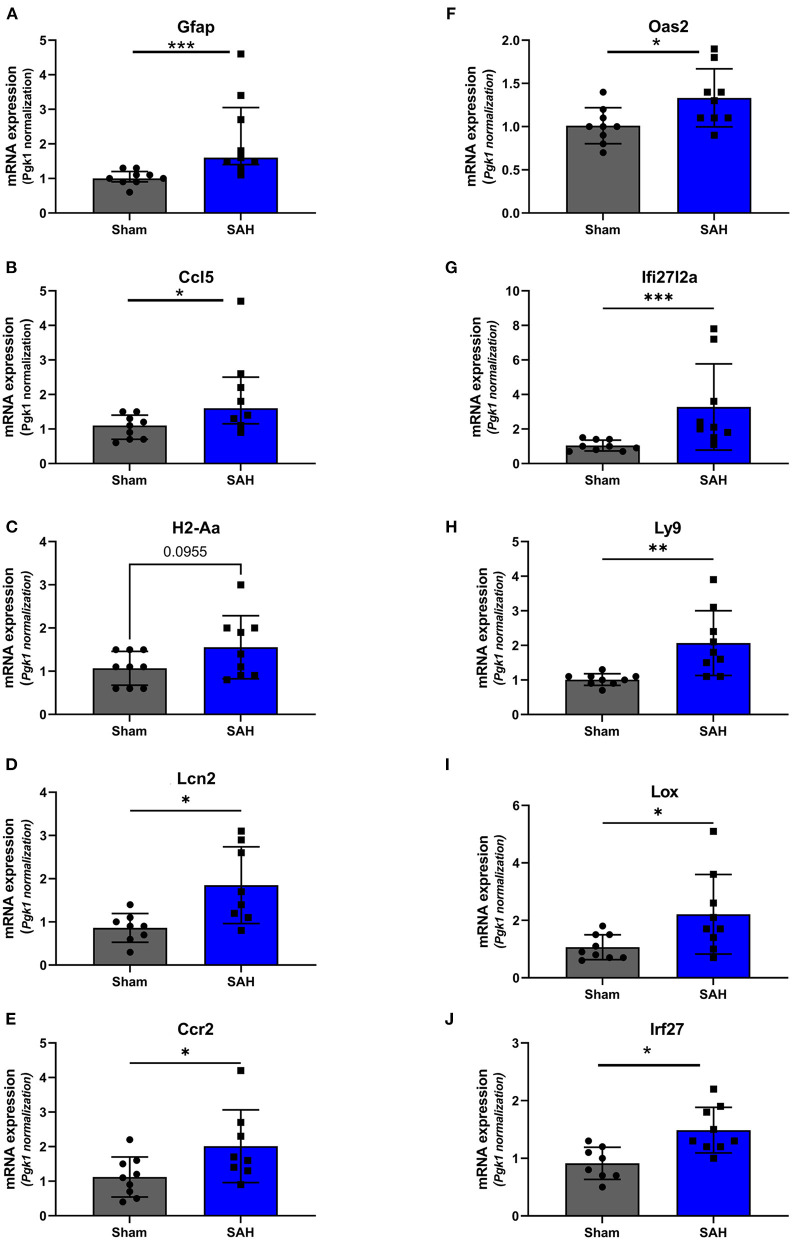
Validation of the RNAseq findings by expression analysis of 10 overexpressed DEGs using Rt-qPCR at 4 days after SAH. Fold change, calculated by 2^−ΔΔCt^*V*, of mRNA expression of **(A)** GFAP: *U* = 4, *P* = 0.0006; **(B)** Ccl5: *U* = 14.50, *P* = 0.0379 **(C)**. H2-Aa: *t*_(16)_ = 1.772, *P* = 0.0955; **(D)** Lcn2: *t*_(14)_ = 2.940, *P* = 0.0108; **(E)** Ccr2: *t*_(15)_ = 2.20, *P* = 0.0439; **(F)** Oas2: *t*_(16)_ = 2.247, *P* = 0.0264; **(G)** Ifi27l2a: *U* = 3.500, *P* = 0.0004; **(H)** Ly9: *t*_(8.521_) = 3.327, *P* = 0.0095; **(I)** Lox: *t*_(9.547)_ = 2.364, *P* = 0.0408; **(J)** Irf27: *t*_(15)_ = 3.247, *P* = 0.0037. *P < 0.05; **P < 0.01; ***P < 0.0001. *N* = 18, with *n* = 9 for SAH, *n* = 9 for Sham.

**Figure 2:**
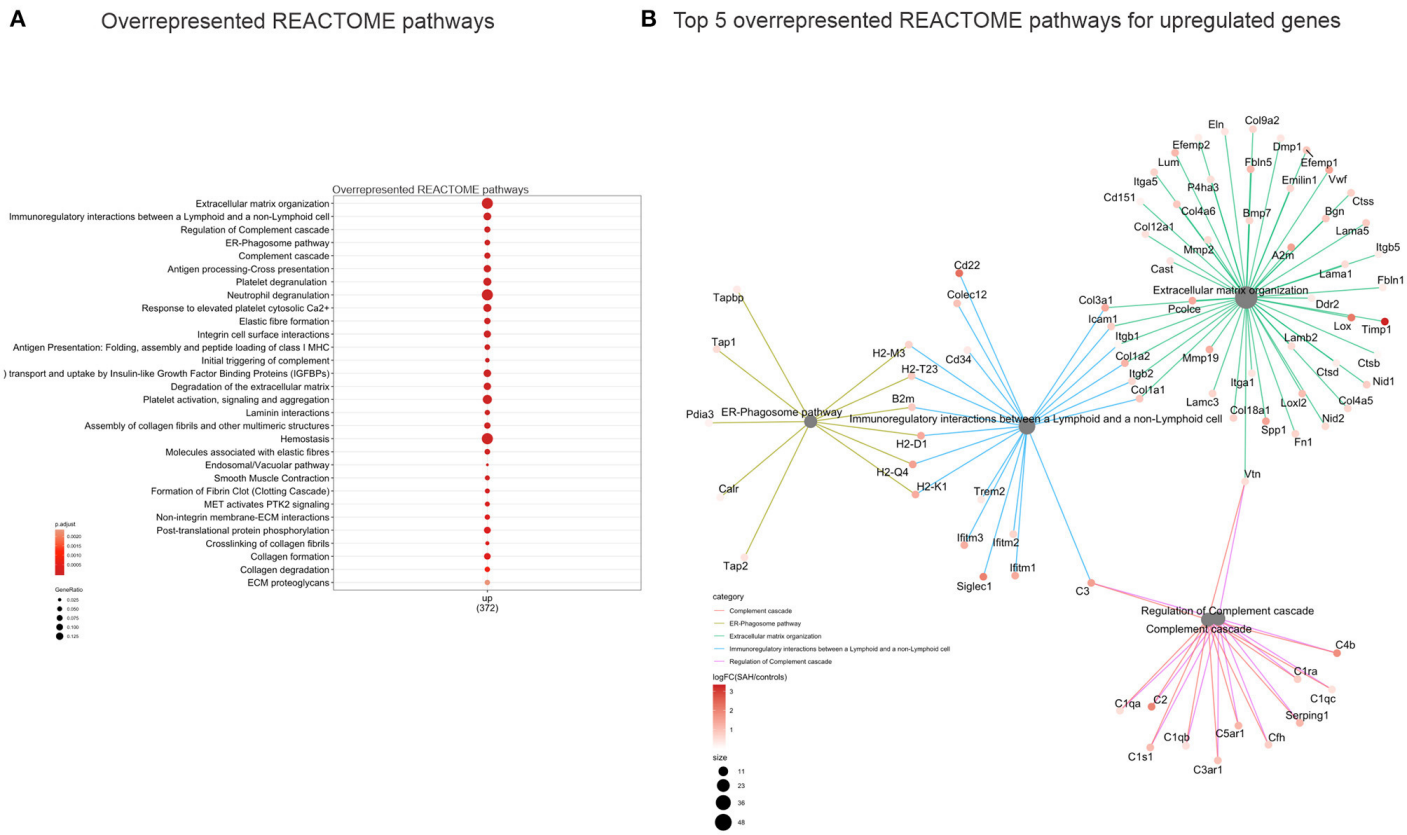
Overrepresented REACTOME pathways 4 days after SAH. **(A)** Dot plot (gene ratio = genes related to a given GO term/total number of DEGs) of the overrepresented REACTOME pathways of all upregulated genes. Note the overrepresentation of immune response-related processes such as “*Extracellular matrix organization*,” “*Immunoregulatory interactions between a Lymphoid and a non-Lymphoid cell*,” and “*ER phagosome pathway*.” **(B)** Interconnected clusters of the five top overexpressed REACTOME pathways. Nodes represent individual genes belonging to a given gene set (or central node), and edges indicate which gene set it belongs to.

**Figure 3:**
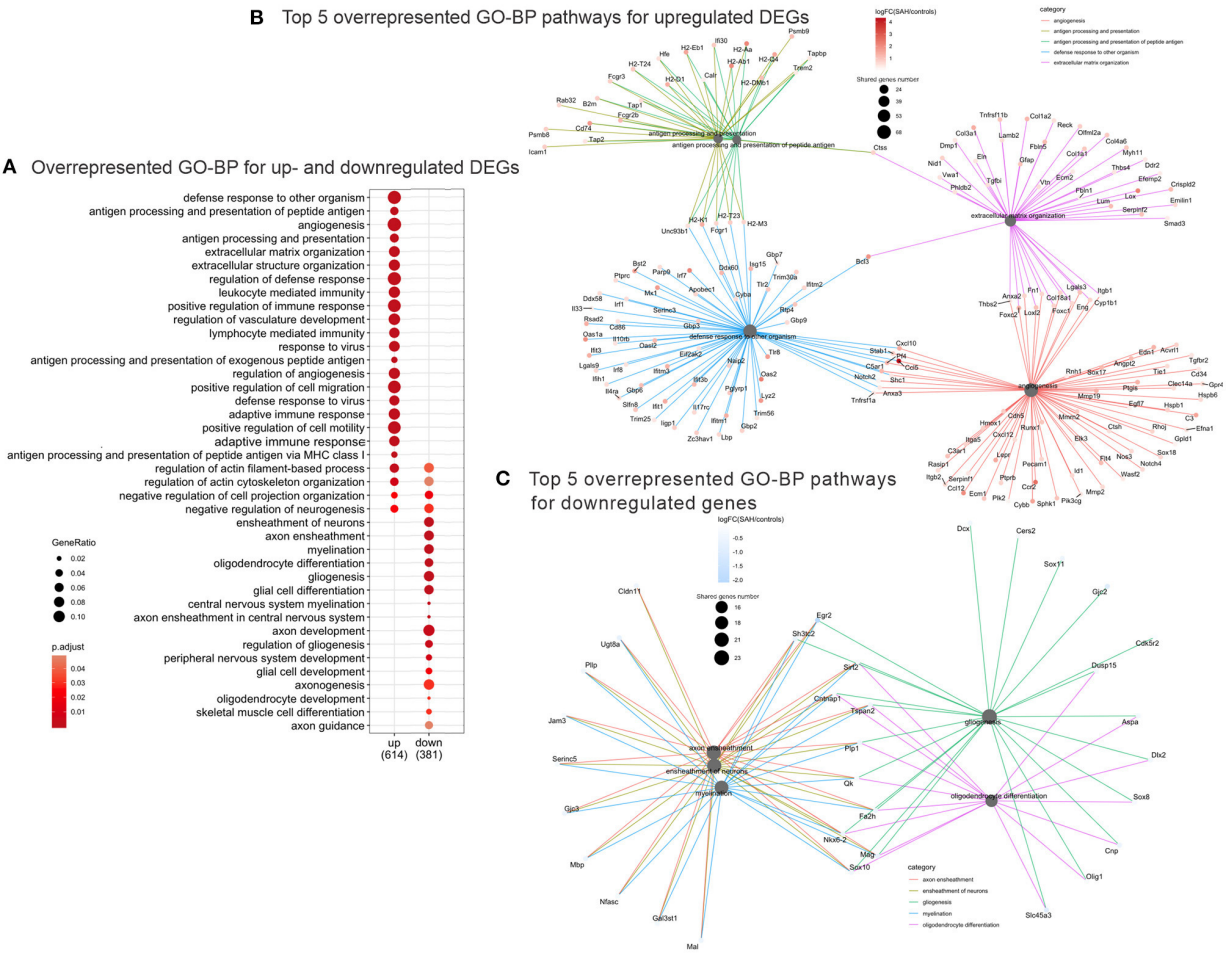
Overrepresented GO Biological Processes 4 days after SAH. **(A)** Dotplot of the most enriched GO biological processes terms for up- and downregulated gene plot. Note that majority of overrepresented biological processes relate to immune response genes (“*immune response*,” “*regulation of vasculature development*,” “*response to virus*,” “*adaptive immune response*,” and “*lymphocyte mediated immunity*”), while the downregulated GO Biological Processes are myelin/axon/oligodendrocytes-related (“*axon ensheathment*,” “*ensheathment of neurons*,” “*myelination*,” “*oligodendrocyte differentiation*,” and “*gliogenesis*”). Color represents adjusted *p*-value or FDR, gene ratio = genes related to a given GO term/total number of DEGs. **(B)** Interconnected clusters of the five top overrepresented GO Biological Processes terms for upregulated genes. Nodes represent individual genes belonging to a given gene set (or central node), and edges indicate which gene set it belongs to. Gene number reflects number of downregulated genes in the central node. **(C)** Interconnected clusters of the five top overrepresented GO Biological Processes terms for downregulated genes. Nodes represent individual genes belonging to a given gene set (or central node), and edges indicate which gene set it belongs to. Color represents adjusted *p*-value or FDR. Gene number reflects number of downregulated genes in the central node.

**Figure 4:**
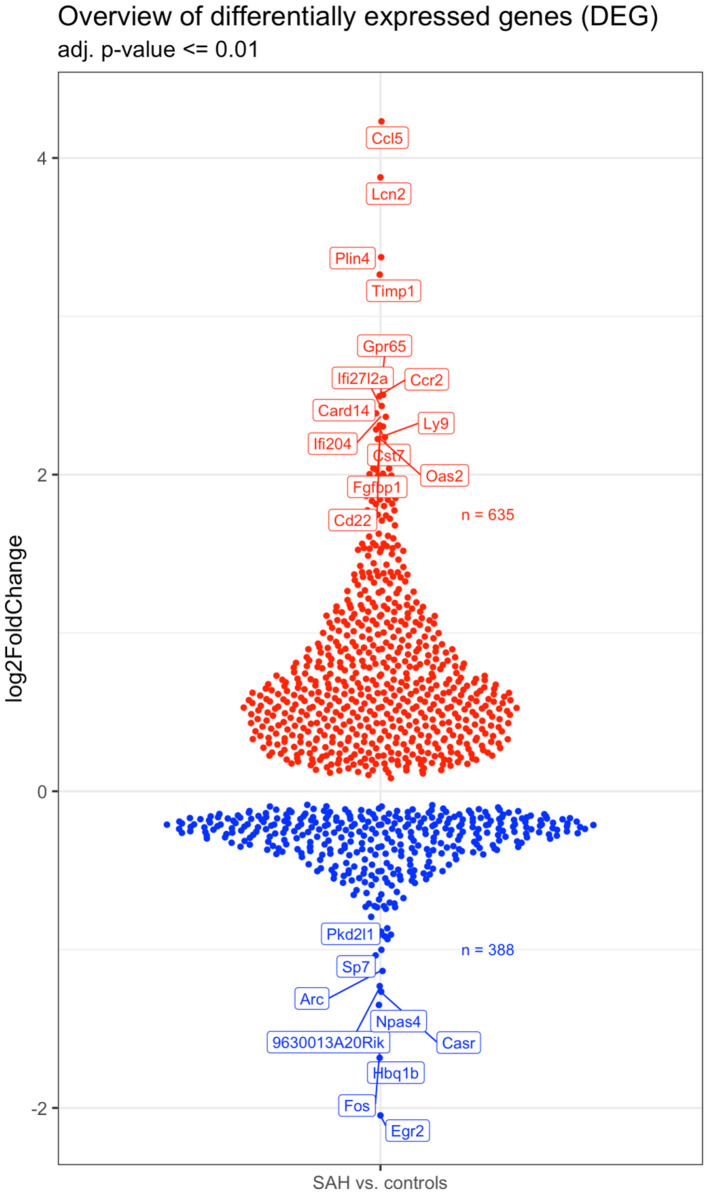
Overview of the 642 upregulated (in red) and 398 downregulated (in blue) genes in the SAH vs. the control samples. The names of the topmost up- and downregulated genes are indicated. *N* = 10 with *n* = 4 for SAH, *n* = 3 for Sham, *n* = 3 for Naïve.

Validation of RNAseq findings was performed with mRNA expression analysis using RtqPCR. We found 10 overexpressed DEGs genes statistically significant in a different cohort of animals (Figure 1). To explore active ongoing processes in the hippocampus, we analyzed DEGs using REACTOME pathways and GO-biological processes (GO-BP) analyses and searched for clusters of interrelated processes. The five top interacting clusters identified, using both approaches, revealed overexpression of groups of genes related to immune and inflammatory processes and extracellular matrix reorganization (Figures 2, 3). Additional exploration using Ingenuity Pathway Analysis canonical pathways (IPA, QIAGEN, 2020) and GSEA Hallmark collection confirmed the prevalence of these processes (Supplementary Figure 3). Five top upregulated genes included *Ccl5, Lcn2, Plin4, Timp1*, and *Gpr65* related to inflammation (Figure 4; Table 1).

**Table 1:** Top 10 upregulated genes potentially associated with different cellular types from single-cell transcriptomics studies.

**Gene symbol**	**Gene name**	**Log_**2**_-fold change**	***padj* value**	**Cell origin (57)**	**Cell origin (58)**
*Ccl5*	C-C motif chemokine 5	4.23	3.2 × 10^−7^	Microglia	T cells, NK/NK T cells
*Lcn2*	Neutrophil gelatinase-associated lipocalin	3.87	0.004	Endothelial	Neutrophils
*Plin4*	Perilipin-4	3.37	0.005	NA	NA
*Timp1*	Metalloproteinase inhibitor 1	3.26	0.0003	Endothelial	NA
*Ccr2*	C-C Chemokine receptor type 2	2.50	0.002	Microglia, OPC	BAM, cDC, Monocytes, Tcells, and NK/NK T cells
*Gpr65*	Psychosine receptor	2.49	0.0003	Microglia	BAM, NK/NK T cells
*Ifi27l2a*	Interferon alpha-inducible protein 27-like protein2	2.43	5.5 × 10^−9^	Microglia	BAM, cDC, B cells, Monocytes, and T cells
*Card14*	Caspase recruitment domain containing protein 14	2.38	0.007	NA	NA
*Ifi204*	Myeloid cell nuclear differentiation antigen	2.36	6.2 × 10^−9^	Microglia	BAM, monocytes
*Cd22*	B-cell receptor	2.31	0.002	Microglia	T cells, NK/NK T cells

**Figure 5:**
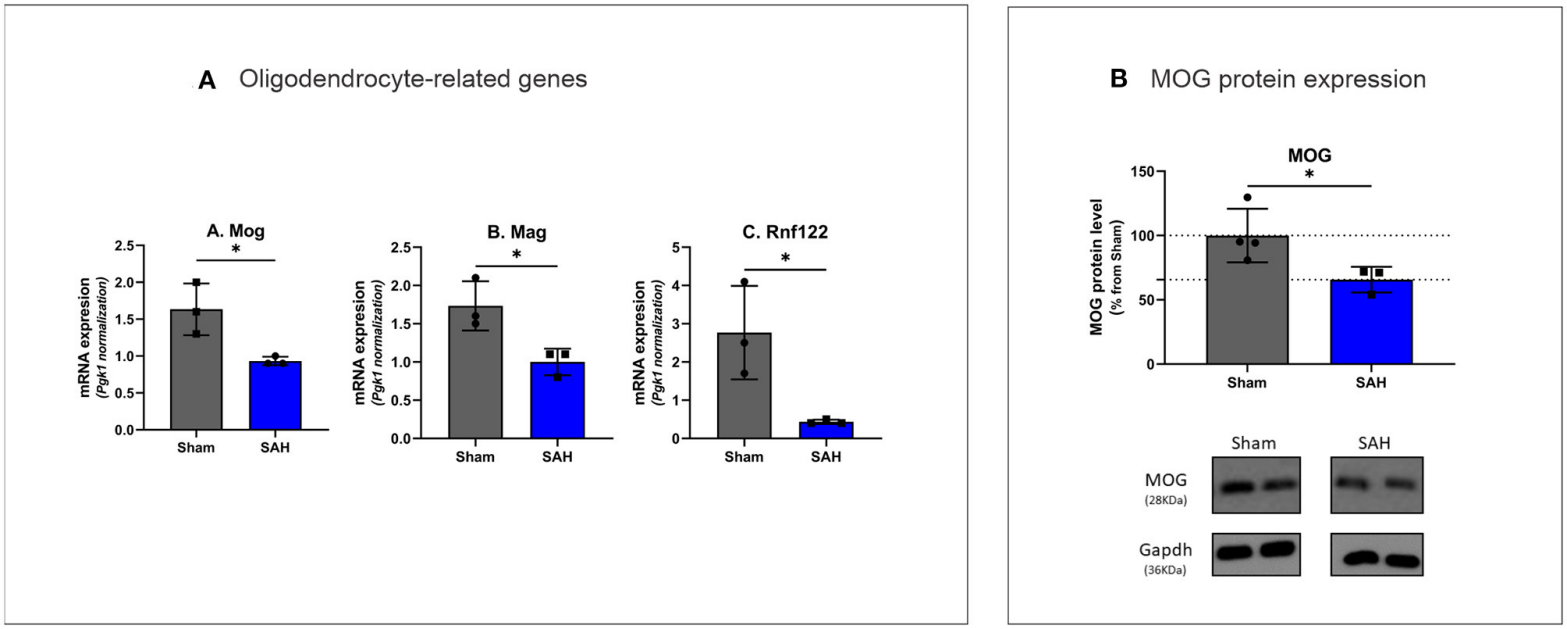
**(A)** Rt-qPCR analysis of oligodendrocyte specific gene expression 4 days after SAH Mog: *t*_(4)_ = 3.407, *P* = 0.0271, Mag: *t*_(4)_ = 3.479, *P* = 0.0254, Rnf122: *t*_(4)_ = 3.304, *P* = 0.0298. *P < 0.05. *N* = 18, with *n* = 9 for SAH, *n* = 9 for Sham. **(B)** Western blot analysis of myelin oligodendrocyte glycoprotein with MOG protein expression decreased by 34.31% in SAH group *t*_(5)_ = 2.590, *P* = 0.0488. *N* = 7, with *n* = 3 for SAH, *n* = 4 for Sham.

The top five downregulated interacting clusters of GO-BP related to myelin ensheathment and oligodendrocyte activity (Figure 3C). Significantly decreased was the expression of genes specific to the myelin structure: *Pllp, Mbp, Mal, Mag, Cntnap1, Plp1, Mog*, and *Mobp* as well as the oligodendrocytes signature genes: *Olig1, Sox8, Sox10*, and *Nkx6.2* (Supplementary Table 3). Decreased levels of the mRNA of *Mog, Mag*, and *Rnf122*, oligodendrocytes-related genes by Rt-qPCR and decrease in MOG protein expression by Western blot (Figures 5A,B) confirmed the decrease of oligodendrocyte activity.

### Immune Response

Over-expression of DEGs of GO-BP suggested activation of genes related to innate and adaptive immunity (Figure 3A). In addition, IPA and GSEA (Supplementary Figures 3A,B) processes demonstrated significant activation of the complement system with activation of the genes coding for the key elements of the complement system *C1, C2, C3, C4*, and *Cfb* and complement system regulators *Cfh* and *C1-inh* (*Serping1*) (Supplementary Table 4). Components of complement receptors CR3 (Cd11b+CD18) and CR4 (Cd11c+CD18), integrins alpha *Itga1, 5* and integrins beta *Itgb1, 2* (CD18), and *5* were also overexpressed. Expression of the gene comprising “membrane attack complex” (*C5-C9*) did not change significantly.

Increased expression of genes belonging to IPA canonical pathways “*Dendritic cell maturation*” and “*Th2 pathway*” suggested activation of adaptive immunity processes (Supplementary Figure 3A). These pathways share class I major histocompatibility complex (MHCI)-related genes, *H2-D1, H2-K1*, and β*2m* (Supplementary Table 5) with the “*Immunoregulatory interactions between a Lymphoid and a non-Lymphoid cell*” REACTOME (Figures 2A,B) along with “A*ntigen presentation*: *folding, assembly*, and *peptide loading of class I MHC*” pathways (Figure 2A). Genes associated with major histocompatibility II (MHCII) *Cd74, H2-Aa, H2-Ab1*, and *H2-Eb1* were also upregulated as well as the costimulatory molecule gene *CD86* (Supplementary Table 6). Chemokine genes involved in macrophage/monocyte recruitment *Ccl5* (4-log_2_-fold increase; Figure 4; Table 1), *Ccr2* (2.5-log_2_-fold increase; Figure 4; Table 1), and its murine ligand *Ccl12* as well as *Cxcl10* (Supplementary Table 7) were also increased. Toll-like receptors (*TLR*) *2* (0.91-log_2_-fold increase, *P* < 0.01), *8* (1.67-log_2_-fold increase, *P* < 0.06), and 13 (0.83-log_2_-fold increase, *P* < 0.003) did significantly increase 4 days after SAH.

Our data showed upregulation of microglial markers of M1-like pro-inflammatory phenotype, particularly microglial signature genes of phagocytotic processes *Calr, Itgb2, Cd68, Cxcl16, Scara5*, and scavenging receptor gene *Scarf1*, along with IgG Fc receptors *Fcgr2b (CD32), Fcgr4 (CD16)*, lectin *Lgals3*, and *Trem2* and its ligand *Tyrobp* (Supplementary Table 8). M2-like anti-inflammatory phenotype *Il14ra, Il13ra1*, and *Il10rb* and M0-resting-state microglia along with upregulation of the growth factors supporting damage repairment and tissue remodeling *Cxcl10, Igf1, Tgfbr2, Tgfbr3, Pdgfd*, and *Mmp2* (Supplementary Table 8) were also increased.

### Upstream Regulators

IPA upstream regulator analysis (URA) showed activation of five top gene sets regulated by IFNG, IRF7, IRF3, STAT1, and CHUK (Supplementary Table 9A). Also, among significantly activated sets of genes were genes under upstream regulators TNF-α, IL1-β, TLR4, and IFNAR1. Supporting the involvement of type I and II interferons' pathways at 4 days after SAH were the overexpression of interferon-related DEGs *Irf7, Irf1*, and *Irf8* as well as upregulation of *Ifit1, Usp18, Isg15, Ifih1*, and *Unc93b1*(Supplementary Table 10) downstream of interferon regulatory genes.

The URA also identified TGFβ1 as a potential activated upstream regulator (Supplementary Table 9A). Supporting TGFβ pathway engagement 4 days after SAH are the overexpression of bone morphogenetic proteins *Bmp5, Bmp6*, and *Bmp7* in DEGs (Supplementary Table 11), *Smad6* and *Smad7*, inhibitory Smads in the TGFβ intracellular pathway, as well as *Smad3*. Also increased in DEGs were the TGFβ receptors *Tgfbr3* and *Tgfbr2* (Supplementary Table 11).

### Motif Enrichment Analyses

Transcription factor binding motif prediction and enrichment analyses (AME, MEME-suite) confirmed URA identification of interferons as upstream regulators, showing enrichment of IRF7 binding motifs and enrichment of STAT1/STAT2 along with binding motif of SPIB (Spi-B Transcription Factor) (Supplementary Figure 4A).

Analysis of enriched motifs independently of their location within the promoter (CENTRIMO, MEME-suite) suggested enrichment of binding sites for the moderately expressed Krüppel-like factor 3 (KLF3) and SP1 transcription factors (Supplementary Figures 3B,C).

## Discussion

In the present study, we observed overexpression of genes related to an immune/neuroinflammatory response in the mouse hippocampus 4 days after SAH. Functional and master regulator's analysis evidenced for an overexpression of genes related to *an innate and adaptive immune response* with possible involvement of blood-resident cells as well as parenchymal reorganization in the hippocampus at 4 days.

These observations are in line with what has been described in the pathophysiology of stroke as a biphasic mechanism, which has become clear over the years of research (9, 59), with the damaged brain exhibiting an initial detrimental response following the acute injury, and regenerative processes during the recovery phase. However, the present study shed light on the leading pathways and specific gene expression occurring in the hippocampus remotely from the site of the infarct, the entorhinal cortex. We discuss further potential use of this newly found druggable targets and potential drug repurposing.

There are some limitations of the study. First is the limited number of animals, which may affect to some degree the significance of the gene expression changes. We used cutoff for DEGs of 0.1, which left out a large number of genes demonstrating sizable increase in expression. Second, the only time point, 4 days, chosen because this is the point when SAH-induced delayed cerebral ischemia (DCI) starts to occur (23, 24). These events have not been well characterized and need further investigations. Additional time points, e.g., 24 h and 30 days, would be useful to understand the dynamics of ongoing processes.

Specifically, we unveiled involvement of the complement in SAH 4 days after the infarct. Genes related to the classical and alternative pathways of the complement system (60) were significantly overexpressed (Supplementary Table 4). These observations are in line with a growing body of evidence demonstrating the importance of the complement activation in neuroinflammation, synaptic pruning, crucial for the establishment of healthy neuronal connections, in neural development and aging (60). Opsonized elements undergo phagocytosis, as reflected in the increased expression of endoplasmic reticulum phagosome pathway and overexpression of signature genes of phagocytosis *Calr, Itgb2, Cd68, Cxcl16*, and *Scara5* (61), currently found in this study. C1 binding initiates classic pathway of complement system activation leading to generation of complement-related opsonins. However, we did not observe increase in lectin pathway genes, nor did we find significant change in the “membrane attack complex,” excluding the complement role in the cell death at least 4 days post-injury. Complement-targeted drugs benefit from intense investigation for neurological diseases and could be potentially used for SAH patients' survivors (62, 63).

We detected overexpression of genes related to monocytes-derived macrophages infiltrating the brain parenchyma following CNS injury and acquiring macrophages phenotype (64). *CCL5* was the highest upregulated gene in this study. CSF levels of its coded protein, chemokine C-C motif ligand 5, was identified as possible predictor of SAH outcome severity (65). CCL5 can be synthesized by astrocytes, microglia, and oligodendrocytes and often associated with major fiber tracts (66). CCL5 attracts T-cells and monocytes/macrophages (67). *CCR2* was also among the top upregulated genes (Table 1; Figure 4). Monocytes with a high expression of CCR2 are reported to enter the brain after transection of the perforant pathway, while lack of CCR2 prevented the entry of macrophages and T cells into the brain parenchyma (68). We also showed an increase in MHCII related genes, which is known to be involved in the crosstalk between microglia/macrophages and T cells (69). Invasion of the brain parenchyma of blood-resident cells has been extensively shown in stroke (70), but little to no evidence was shown after SAH. Our results indicate that immuno-therapy targeting myeloid cells could be employed in the treatment of SAH (71).

We observed a decrease of genes expression related to axons, myelin, and myelin formation as well as decrease in oligodendrocytes-related genes, which suggest a demyelinating process (Figure 3C; Supplementary Table 3). This process could be mediated by increased expression of *Bmp* and semaphorin genes, which are known to suppress myelination and oligodendrocyte differentiation (72). It was also reported that *Lcn2*, our second top overexpressed gene preferentially localized to the white matter, is activated after SAH and plays an important role in damaging oligodendrocytes (73). Silencing or blocking *Lcn2* could protect oligodendrocytes viability and engage in a faster remyelination process (74).

The gene expression profile of microglia-related genes presented here is comparable to demyelination/remyelination processes at the end of 6 weeks in cuprizone multiple sclerosis model (61) with M1, M2, and M0 signature genes (Supplementary Table 8). Myelin clearance following axonal damage is crucial as the presence of myelin debris inhibits the processes of regeneration and repair (75). Expression of *TREM2* and its ligand *Tyrobp* were significantly increased in the present study. TREM2 has been shown to play a significant role in Alzheimer's disease (76). TREM2 microglial receptor is critical for efficient removal of myelin debris after cuprizone-triggered demyelination (77), and other genes related to phagocytosis, such as *Fc*γ*R3, CD68*, and *Lamp2*, were also significantly overexpressed (Supplementary Table 12) (78). Modulation of TREM-2 could accelerate myelin debris clearance and promotes repair (79, 80).

Mutually promoting relations between vessels and axon growth are known (81): blood vessels guide developing axons by secretory factors (82) and form a migratory scaffold for neuroblast in post-stroke area (83). Pleiotropic TGFβ/BMP pathways, identified by URA, can exert a dual pro- and anti-inflammatory action on the CNS (84) and may participate in the regenerative processes such as scar formation and angiogenesis (85). Our functional analysis detected an overrepresentation of the GSEA pathways “*Epithelial_Mesenchymal_Transition*” and “*Angiogenesis*” pathways (Supplementary Figure 3B), as well as GO-BP terms “*angiogenesis*” and “*extracellular matrix and structure organization*” (Figure 3A). These data suggest that the relation between EC-OPCs and role of TGFβ superfamily deserves more investigation after SAH.

IPA URA and findings of the enrichment of DNA binding motif suggested activation of interferon-pathway related genes (Supplementary Table 9A). These results were supported by the GSEA analysis that detected an enrichment in the “*interferon-*γ *response*” and “*interferon-*α *response*” gene-sets (Supplementary Figure 3B). It is known that activation of TLRs regulates immune response through MyD88 or TRIF pathways and leads to activation of interferon regulated transcription factors (IRF) (86). *TLR* 2, 8, and 13 were among overexpressed DEGs, which serve as a pattern recognition receptor and co-regulate antigen processing and presentation (87). TLR 2, located on the cell surface, preferentially binds lipid-containing damage associated molecular patterns (DAMP) and shares intracellular pathways with TLR4. TLR8 and TLR13 are intracellular receptors located in the endosomes and are specific for double-stranded and single-stranded nucleic acid detection (87). In our study, *Irf7, Irf1*, and *Irf8* were significantly overexpressed along with activation of 35 out of 36 genes comprising IRF3 regulated group, including overexpression of interferon-α inducible protein 27 gene (*Ifi27l2a*) (Figure 1). *Irf7* increased by 2.1-log_2_-fold in our DEGs, and our common motif analysis demonstrated its significant presence among our set of DEGs. Irf7 is regulated in a cell-type-dependent manner and is required for a maximal type I IFN-α gene response (88). Irf8 was shown to be an important factor during the recovery phase following spinal cord injury (89). Type I IFN-α and II (IFN-γ) gene responses have been repeatedly shown to be important for brain repair and maintenance (90). IFN-γ is a critical regulator of blood cell entry in the brain at the choroid plexus entry point (90).

Our motif enrichment analysis supported the involvement of adaptive immunity cells and the regeneration process with SPIB (Spi-B Transcription Factor), which plays a critical role in regulation of dendritic cell and B cell development and antigenic stimulation (91). Also enriched was binding motif of FOXO3, which participates in controlling of expression of autophagy related genes (92). KLF3 is a known regulator of B cells (93), and SP1 plays a role in regeneration gene expression (40).

The present transcriptome analysis of the processes in the hippocampal tissue 4 days after SAH suggests the development of an inflammation in the hippocampus remote from the entorhinal cortex. Data suggest that direct application of blood to the brain surface damages neurons of the entorhinal cortex, leading to the damage of the perforant pathway, main projections to the hippocampus (14, 15), and to the hippocampus itself (16–18). This initial event would lead to the gene expression we described and expression of the specific genes involved. Further experimental explorations of the results highlighted here will allow identification of new therapeutic targets to alleviate long-term consequences of SAH.

## Data Availability Statement

The datasets presented in this study can be found in online repositories. The names of the repository/repositories and accession number(s) can be found at: https://www.ncbi.nlm.nih.gov/geo/ (GSE167110); https://github.com/abcwcm/Regnier-Golanov2021 (Regnier-Golanov2021).

## Ethics Statement

The animal study was reviewed and approved by Institutional Animal Care and Use Committee of Houston Methodist Research Institute, Houston, TX.

## Author Contributions

AR-G and EG conceived and designed experiments, acquired, analyzed, interpreted data, and wrote the manuscript. MH, FD, PZ, DB, and LP analyzed and interpreted data and edited the manuscript. EL interpreted data and drafted and critically revised the manuscript. GB conceived the experiments, analyzed data, and drafted and critically revised the manuscript. All authors contributed to the article and approved the submitted version.

## Conflict of Interest

The authors declare that the research was conducted in the absence of any commercial or financial relationships that could be construed as a potential conflict of interest.
