# Supplementary material for: Hippocampal Transcriptome Changes After Subarachnoid Hemorrhage in Mice
Source: Front Neurol. 2021 Jul 20;12:691631. doi: 10.3389/fneur.2021.691631 (PMC8329593; doi:10.3389/fneur.2021.691631)
Supplement: Supplementary file 1 [file Data_Sheet_1.pdf]

Supplementary Material

1 Supplementary Figures and Tables

**Gene ID**	**Forward**	**Reverse**
Pgk1	ATGCTTTTGGGACTGCACAC	AACTTTAGCGCCTCCCAAGA
Gfap	ACTCAATACGAGGCAGTGGC	CTCTAGGGACTCGTTCGTGC
Ccl5	GACAGCACATGCATCTCCCA	GTGTCCGAGCCATATGGTGA
H2-Aa	CCAGCTACCAATGAGGCTCC	CGTCTGCGACTGACTTGCTA
Lcn2	CCACCACGGACTACAACCAG	TCCTTGGTTCTTCCATACAGGG
Ccr2	AAGGAGCCATACCTGTAAATGC	GCCGTGGATGAACTGAGGTAA
Oas2	TGAAGACCGTCAAGGGAGGA	TCGTAACTCTCCAGCGAGGA
Ifi27l2a	CACTCCAATCAGCAGGGGTCC	CAGTGAGGGTTCTGAAGGTGC
Ly9	ATTCTCGCCGTAAGCCCAAA	GGGCTTCTCCAGTCTCTTGTAG
Lox	ACTGCACACACACAGGGATT	TGTAGCGAATGTCACAGCGT
Irf7	CAATGGCTGAAGTGAGGGGG	TCCAGATCCCTACGACCGAA

Supplementary Table 1: Primers used for Rt-qPCR overexpression validation

Day		Group (mean±SD)		Between groups
	**Naïve (n=3)**	**Sham (n=3)**	**SAH (n=4)**	**Kruskal-Wallis-test**
Preoperative	17±0	17.66±0.57	17.25±0.5	$H_{(3)}$=2.929, p=0.400
24h	17.33±0.57	14.67±3.21	15.75±1.89	$H_{(3)}$=3.581, p=0.1976
48h	17.33±0.57	15.67±1.53	16.5±11.29	$H_{(3)}$=3.613, p=0.1581
72h	17.67±0.57	16.33±0.57	16.75±0.95	$H_{(3)}$=3.653, p=0.1971
96h	17.33±0.57	16.33±0.57	17.25±0.5	$H_{(3)}$=4.313, p=0.1857

Supplementary Table 2: Garcia score. N=10 with n=4 for SAH, n=3for Sham, n=3 for Naïve.

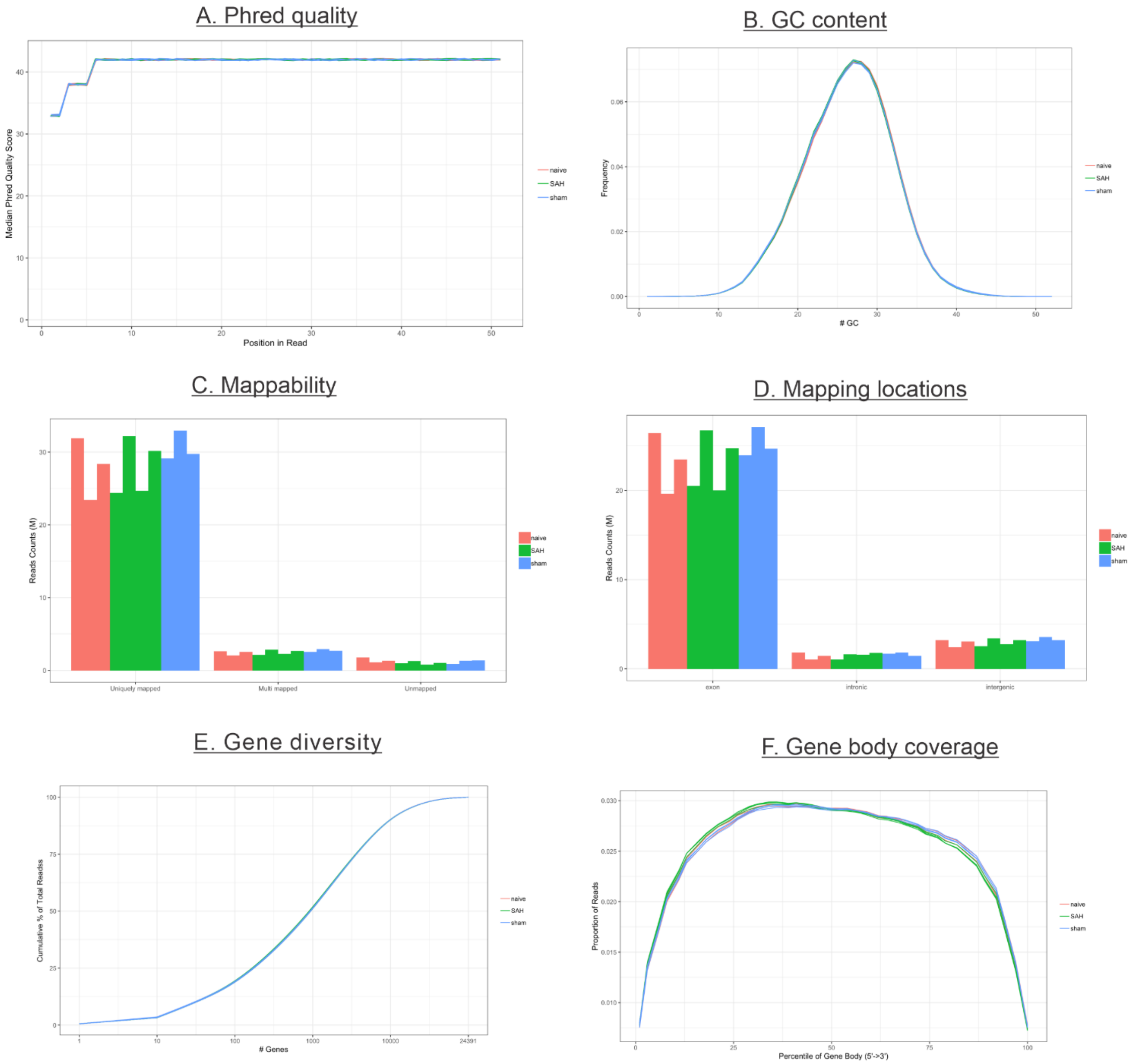

<u>Supplementary Figure 1</u> QC plots from FastQC and QoRts of our 10 samples, each consisting of 4 or 3 biological replicates of 3 conditions (SAH: n=4; Sham: n=3; Naïve: n=3). Colors apply to biological groups (**A)** All reads in the 10 samples reached a maximum Phred score of 40 after the 6th nucleotide, corresponding to a 99% accuracy in the base identification (**B)** The GC content of all reads displays a normal distribution over all sequences as in normal random libraries (**C)** Our RNA sequencing achieved a minimum depth of 20-30million reads per sample uniquely mapped to the genome matching Encode guidelines (**D)** Our sequences mRNAs have little to no reads mapping the intronic or intergenic regions (**E)** The cumulative gene diversity shows our sequencing complexity

with 100% of the reads representing 24391 genes, with no indication that a small subset of genes is over-represented in any replicate (**F**). The gene-body coverage profile for each replicate shows relatively uniform coverage across quantiles of all gene’s lengths, form 5’ to 3’

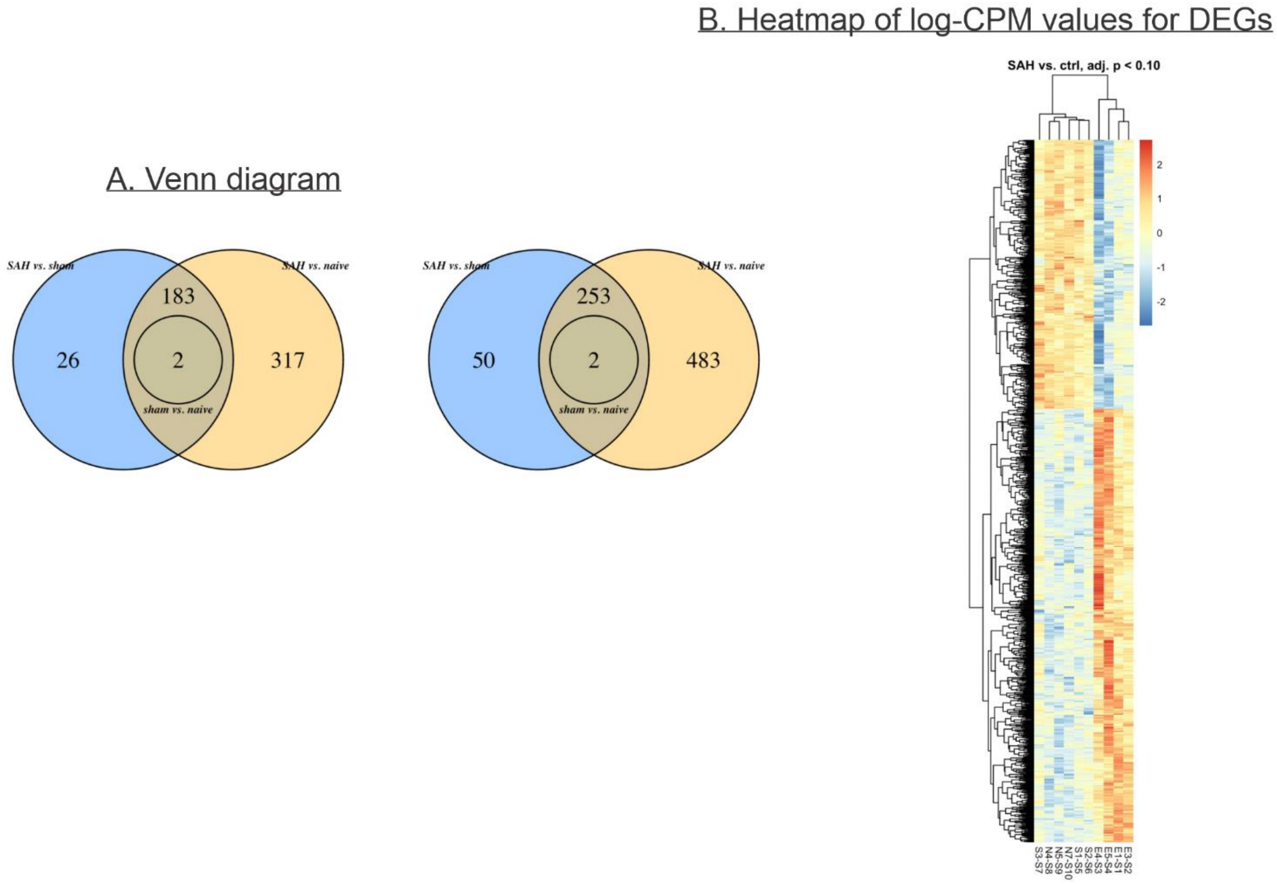

Supplementary Figure 2 (**A)** Venn diagram of the Wald test showing the overlap between the 3 pairwise comparisons, and that only 2 genes differ between Sham and Naïve for FDR<0.10 (**B**) Heatmap of the 1040 differentially expressed genes (FDR<0.1) indicated as log2 counts-per-million and scaled by row. E-SAH, S-Sham, N-Naïve. N=10 with n=4 for SAH, n=3for Sham, n=3 for Naïve.

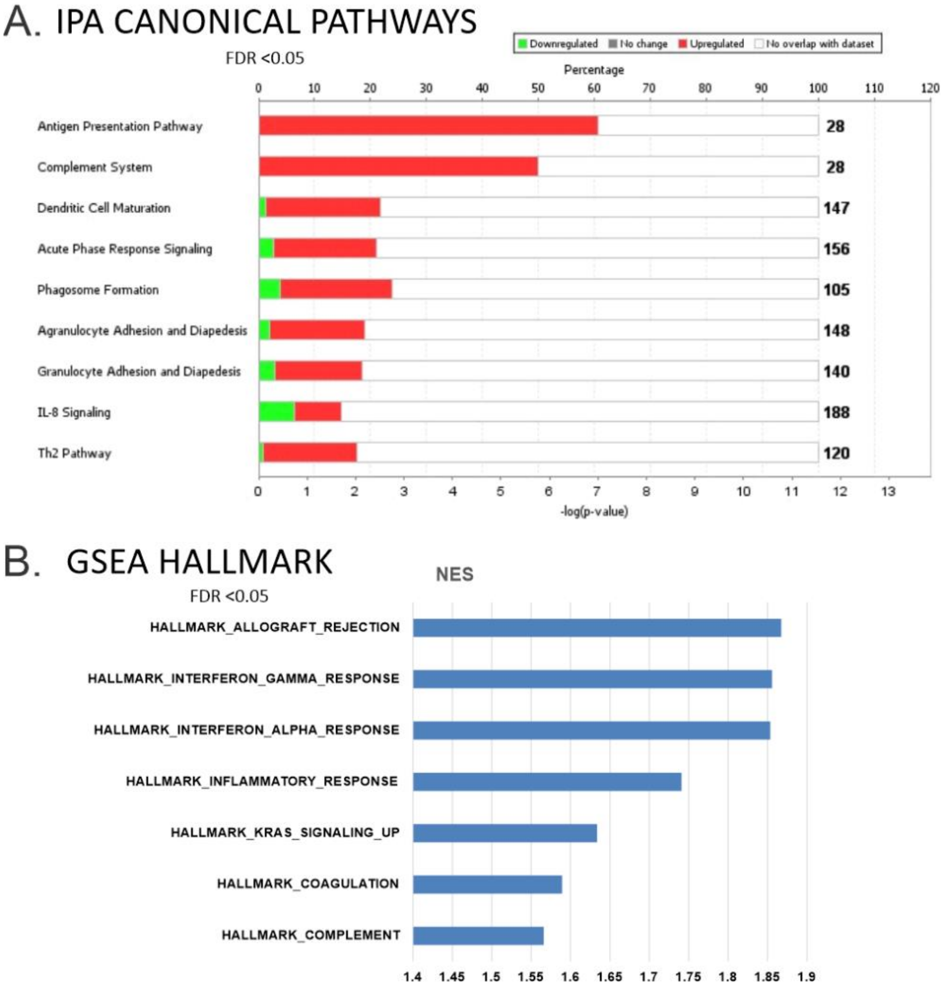

Supplementary Figure 3 (**A)** Canonical pathways identified by Ingenuity Pathway Analysis (IPA) of DEGs 4-days after SAH. Bar plots of the top 10 canonical IPA pathways. Bars represent percentage of DEGs present in canonical IPA with red being the up-regulated genes and green the down-regulated genes in each pathway significantly changed (**B**) GSEA_Hallmark MsigDB analysis of post-SAH overexpressed DEGs. Bar plots from gene set enrichment analysis with the highest Normalized Enrichment Score (NES)

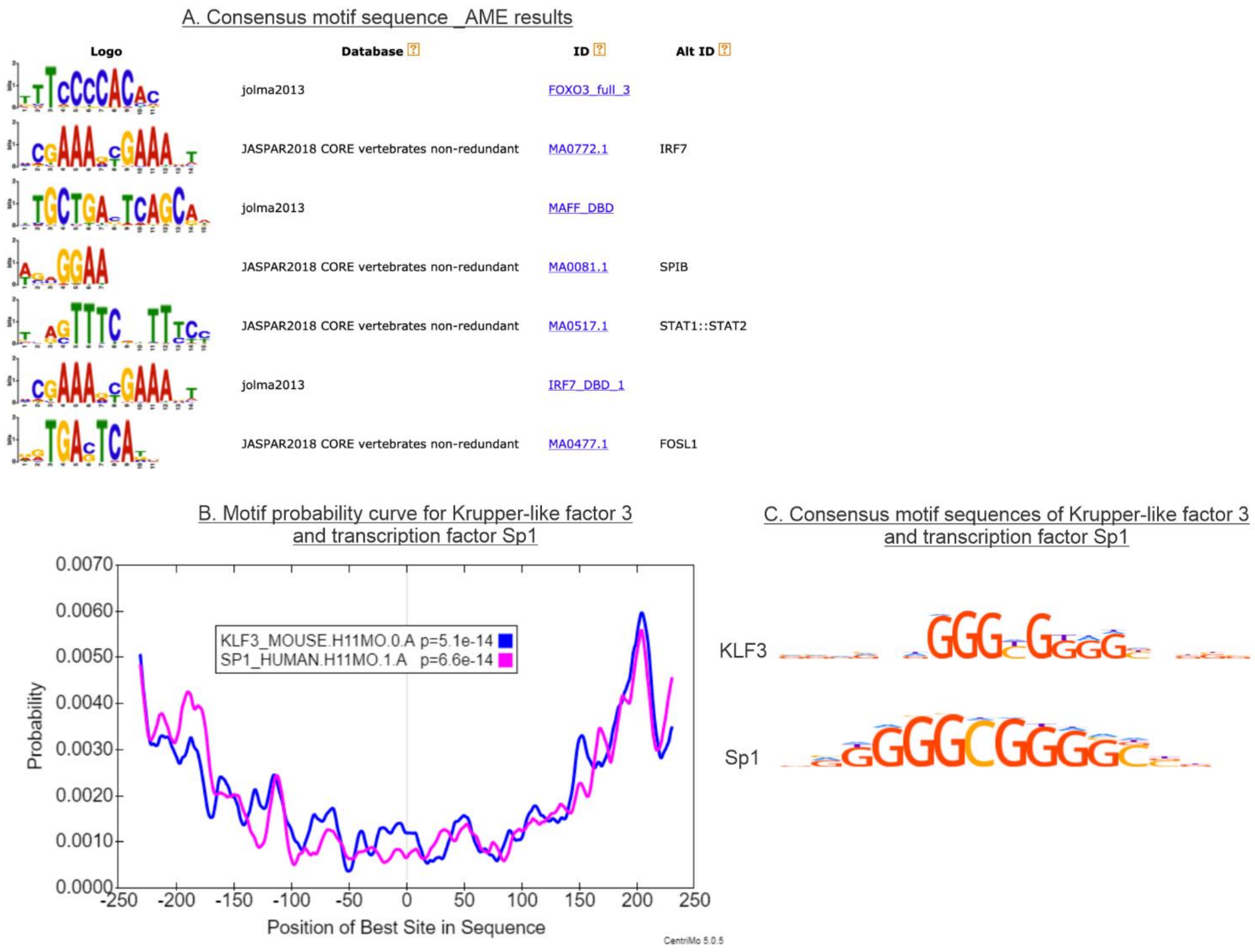

Supplementary Figure 4 Identification of common DNA motifs significantly present in DEG set

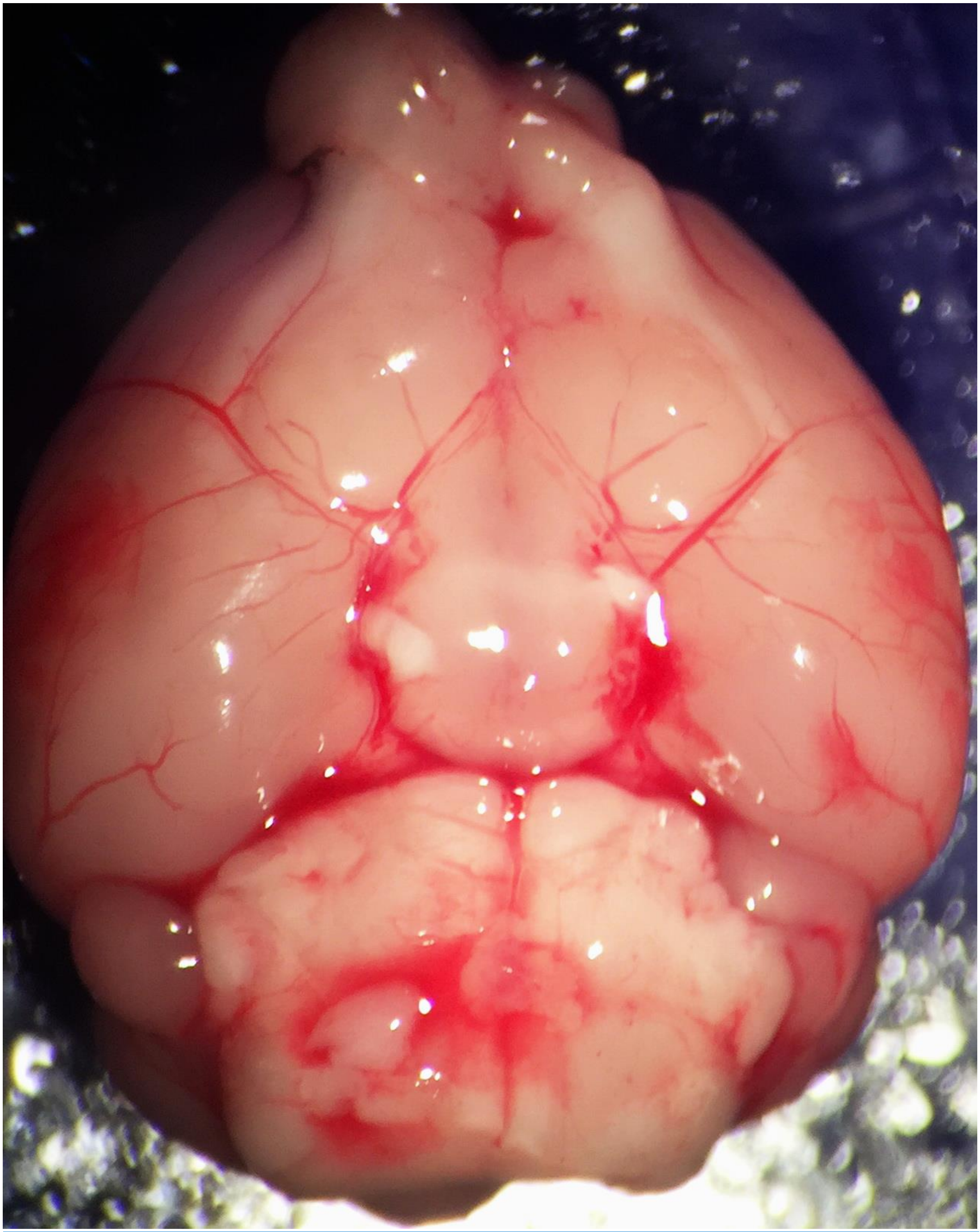

Supplementary Figure 5 Photograph of the brain ventral view of a representative SAH animal 4-days after the surgery in the present study

Gene symbol	Gene name	Log2 (Fold change)	*padj* value
Mog	Myelin basic protein	-0.72	2.38 10^{-8}
Mag	Myelin-associated glycoprotein	-0.67	3.47 10^{-10}
Mbp	Myelin basic protein	-0.59	2.38 10^{-8}
Plp1	Myelin proteolipid protein	-0.55	4.76 10^{-6}
Mal	Myelin and lymphocyte protein	-0.52	9.86 10^{-7}
Nkx6.2	NK6 homeobox 2	-0.52	0.005
Mobp	Myelin-associated oligodendrocyte basic protein	-0.5	0.0001
Pllp	Plasmolipin	-0.46	0.0007
Sox10	Transcription factor Sox-10	-0.4	0.06
Sox8	Transcription factor Sox-8	-0.27	0.07
Olig1	Oligodendrocyte transcription factor	-0.183	0.09
Cntnap1	Contactin-associated protein 1	-0.124	0.06

Supplementary Table 3 Expression changes of oligodendrocytes-related genes in mouse hippocampus 4 days following SAH

Gene symbol	Gene name	Fold change	*padj* value
Cfb	Complement factor B	2.17	$7.04\ 10^{-3}$
C2	Complement C2	1.97	$6.21\ 10^{-7}$
C4	Complement C4	1.81	8.29 10-^{9}
C3	Complement C3	1.53	$5.49\ 10^{-5}$
C5ar1	C5a anaphylatoxin chemotactic receptor 1	1.19	$9.00\ 10^{-2}$
Serping1	Plasma protease C1 inhibitor	1.17	$1.52\ 10^{-19}$
C1s	Complement C1s	1.09	$2.77\ 10^{-3}$
C3ar1	C3a anaphylatoxin chemotactic receptor 1	0.98	$2.29\ 10^{-5}$
Cfh	Complement factor H	0.81	$3.57\ 10^{-8}$
C1r	Complement C1r	0.79	$2.37\ 10^{-2}$
Itgb2	Integrin subunit beta 2	0.62	$3.73\ 10^{-2}$
C1qa	Complement C1q subcomponent subunit A	0.52	$5.42\ 10^{-5}$
C1qb	Complement C1q subcomponent subunit B	0.48	$3.23\ 10^{-5}$
C1qc	Complement C1q subcomponent subunit C	0.47	$4.15\ 10^{-5}$

Supplementary Table 4 Expression changes of complement system related genes in mouse hippocampus 4 days following SAH

**Gene symbol**	**Gene name**	**Log2 (Fold change)**	***P adj* value**
H2-Q4	Histocompatibility 2, Q region locus 4	1.52	$1.17\ 10^{-7}$
H2-D1	H-2 class I histocompatibility antigen, D-P alpha chain	1.38	$4.91\ 10^{-6}$
H2-K1	H-2 class I histocompatibility antigen, A-K alpha chain	1.35	$1.94\ 10^{-8}$
H2-K2	H-2 class II histocompatibility antigen, K-B alpha chain	1.07	0.02
H2-DMb1	Class II histocompatibility antigen, M beta 1 chain	1.06	0.02
H2-T24	Histocompatibility 2, T region locus 24	0.90	0.02
H2-T23	H-2 class I histocompatibility antigen, D-37 alpha chain	0.84	$2.69\ 10^{-6}$
H2-M3	Histocompatibility 2, M region locus 3	0.69	0.06

Supplemental Table 5 Expression changes of MHC-I related genes in mouse hippocampus 4 days following SAH.

**Gene symbol**	**Gene name**	**Log2 (Fold change)**	***P adj* value**
H2-Aa	H-2 class II histocompatibility antigen, A-K alpha chain	2.09	$2.38\ 10^{-7}$
H2-Ab1	H-2 class II histocompatibility antigen, A-D beta chain	2.03	$1.2\ 10^{-5}$
H2-Eb1	H-2 class II histocompatibility antigen, E-B beta chain	1.53	0.0003

Supplemental Table 6 Expression changes of MHC-II related genes in mouse hippocampus 4 days following SAH

Gene symbol	Gene name	Log2 (Fold change)	*padj* value
Ccl12	C-C motif chemokine 12	1.99	0.0009
Ccr1	C-C chemokine receptor type 1	1.68	0.01
Cxcl10	C-X-C motif chemokine 10	1.39	0.045
Cxcl16	C-X-C motif chemokine 16	0.83	0.02
Cxcl12	C-X-C motif chemokine 12	0.57	0.07

Supplementary Table 7 Expression changes of chemokines-related genes in mouse hippocampus 4 days following SAH (Trettel et al., 2019)

Gene symbol	Gene name	Log2 (Fold change)	*padj* value
**M1-proinflammatory**			
Fcgr4	Low affinity immunoglobulin gamma Fc region receptor IV	1.87	0.01
Lyz2	Lysozyme C-2	1.77	$3.09\ 10^{-36}$
Fcgr2b	Fc fragment of IgG receptor IIb	1.17	$2.39\ 10^{-8}$
Scara5	Scavenger receptor class A member 5	1.15	0.07
Lgals3	Galectin-3	1.02	0.03
Scarf1	Scavenger receptor class F member 1	0.92	0.01
Cxcl16	C-X-C motif chemokine 16	0.83	0.02
Itgb2	Integrin-beta 2	0.61	0.03
Cd86	T-lymphocyte activation antigen CD86	0.48	0.09
Cd68	Macrosialin	0.29	0.03
Calr	Calreticulin	2.90	0.06
**M2-anti-inflammatory**			
IL4ra	Interleukin-4 receptor subunit alpha	0.65	0.04
IL13ra1	Interleukin-13 receptor subunit alpha-1	0.58	$6.9\ 10^{-6}$
IL10rb	Interleukin 10 receptor subunit beta	0.45	0.01
**M0-homeostatic**			
Siglec1	Sialic acid binding Ig like lectin	2.03	0.002
**Socs3**	Suppressor of cytokine signaling 3	1.06	0.08
**Stab1**	Stabilin1	0.83	6.510^{-9}
Cd33	Cluster of differentiation 33	0.7	0.001
C1qa	Complement C1q A chain	0.52	5.4210^{-5}

Crybb1	Crystallin beta B1	0.49	0.05
Trem2	Triggering receptor expressed on myeloid cells 2	0.34	0.01
Tyrobp	Transmembrane immune signaling adaptor	0.32	0.02
Csf1r	Colony stimulating factor 1 receptor	0.28	0.001
Itgb5	Integrin subunit beta 5	0.27	0.003
Hexb	Hexosaminidase subunit beta	0.22	0.007

Supplementary Table 8 Expression changes of microglia signature genes of M1-, M2 and M0-resting phenotype genes in mouse hippocampus 4 days following SAH (Zhang et al., 2014; Crotti and Ransohoff, 2016)

Upstream Regulator	Molecule Type	Activation z-score	p-value
IFNG	cytokine	6.431	1.19E-37
IRF7	transcription regulator	6.098	6.88E-20
IRF3	transcription regulator	5.584	5.42E-19
STAT1	transcription regulator	5.456	3.02E-19
CHUK	kinase	4.713	9.81E-15
TGFB1	growth factor	4.325	1.32E-22
IKBKB	kinase	4.311	1.02E-18
PRL	cytokine	4.111	4.32E-08
IKBKG	kinase	4.1	8.85E-14
IL21	cytokine	4.049	3.85E-10
APP	other	4.004	4.50E-21
TNF	cytokine	3.833	1.14E-28
TICAM1	other	3.804	5.26E-10
IFNAR1	transmembrane receptor	3.794	7.64E-07
IL1B	cytokine	3.706	2.10E-18
SAMSN1	other	3.638	1.32E-05
TLR4	transmembrane receptor	3.543	1.49E-08
DOCK8	other	3.5	1.76E-06
IRF5	transcription regulator	3.388	1.68E-07
TLR7	transmembrane receptor	3.385	1.49E-04

Â© 2000-2020 QIAGEN. All rights reserved.

Supplementary Table 9A Activated upstream regulators in mouse hippocampus 4 days after SAH (Ingenuity Pathway Analysis, QIAGEN)

Upstream Regulator	Molecule Type	Activation z-score	p-value
TCF7L2	transcription regulator	-7.206	3.13E-27
SOX2	transcription regulator	-7	1.89E-24
BDNF	growth factor	-5.032	6.35E-07
PTGER4	G-protein coupled receptor	-4.284	3.59E-12
NRAS	enzyme	-3.952	1.63E-16
SOCS1	other	-3.889	6.16E-10
TSC2	other	-3.696	3.65E-10
Irgm1	other	-3.692	8.81E-08
ZNF106	other	-3.606	3.57E-07
CREM	transcription regulator	-3.563	1.40E-04
NKX2-3	transcription regulator	-3.206	4.04E-11
PSEN1	peptidase	-3.096	1.64E-07
COL18A1	other	-3.032	3.94E-06
B4GALNT1	enzyme	-2.985	1.99E-09
APEX1	enzyme	-2.985	6.11E-09
NCSTN	peptidase	-2.985	6.38E-05
MAPK1	kinase	-2.9	1.44E-04
APOE	transporter	-2.781	1.29E-03
GFI1	transcription regulator	-2.673	7.15E-03
ST8SIA1	enzyme	-2.63	7.84E-07
Â© 2000-2020 QIAGEN. All rights reserved.			

<u>Supplementary Table 9B</u> Inhibited upstream regulators in mouse hippocampus 4 days after SAH (Ingenuity Pathway Analysis, QIAGEN)

**Gene symbol**	**Gene name**	**Log2 (Fold change)**	***padj* value**
Ccl5	C-C motif chemokine 5	4.23	$3.2\ 10^{-7}$
Irf7	IFN-regulatory factor 7	2.1	$2.72\ 10^{-13}$
Mx1	MX dynamin like GTPase 1	1.72	0.02
Usp18	Ubiquitin specific peptidase 18	1.71	$7.21\ 10^{-5}$
Ifit1	Interferon-induced protein with tetratricopeptide repeats 1	1.41	$1.58\ 10^{-8}$
Isg15	ISG15 ubiquitin like modifier	1.26	0.007
Irf1	IFN-regulatory factor 1	0.57	0.0008
Ifi27	Interferon alpha inducible protein 27	0.52	$4.39\ 10^{-7}$
Unc93b1	Protein unc-93 homolog B1	0.5	$2.29\ 10^{-5}$
Irf8	IFN-regulatory factor 8	0.49	0.02

Supplemental Table 10 Expression changes of type I IFN response-related genes in mouse hippocampus 4 days after SAH

Gene symbol	Gene name	Log2 (Fold change)	*padj* value
Bmp5	Bone morphogenetic protein 5	0.9	0.05
Bmp6	Bone morphogenetic protein 6	0.9	$4.97\ 10^{-5}$
Smad6	SMAD family member 6	0.7	0.05
Bmp7	Bone morphogenetic protein 7	0.67	0.04
Tgfbi	Transforming growth factor-beta-induced protein ig-h3	0.51	0.02
Tgfbr3	TGF-beta receptor type-3	0.51	0.008
Tgfbr2	TGF-beta receptor type-2	0.41	0.002
Smad3	SMAD family member 3	0.29	0.0001
Smad7	SMAD family member 7	-0.38	0.001

Supplemental Table 11 Expression changes of TFG-β-related genes in mouse hippocampus 4 days after SAH

**Gene symbol**	**Gene name**	**Log2 (Fold change)**	***padj* value**
Cr3	Complement receptor 3	0.98	2.29 10^{-5}
FcγR3	Fc gamma Receptor 3	0.78	1.69 10^{-5}
Cd68	Macrosialin	0.29	0.03
Lamp2	Lysosome-associated membrane	0.20	0.012

Supplemental Table 12 Expression changes of microglia's phagocytosis genes under IFN-β's regulation in mouse hippocampus 4 days following SAH

Crotti, A., and Ransohoff, R.M. (2016). Microglial Physiology and Pathophysiology: Insights from Genome-wide Transcriptional Profiling. *Immunity* 44(3)**,** 505-515. doi: 10.1016/j.immuni.2016.02.013.

Trettel, F., Di Castro, M.A., and Limatola, C. (2019). Chemokines: Key Molecules that Orchestrate Communication among Neurons, Microglia and Astrocytes to Preserve Brain Function. *Neuroscience*. doi: 10.1016/j.neuroscience.2019.07.035.

Zhang, Y., Chen, K., Sloan, S.A., Bennett, M.L., Scholze, A.R., O'Keeffe, S., et al. (2014). An RNA-sequencing transcriptome and splicing database of glia, neurons, and vascular cells of the cerebral cortex. *J Neurosci* 34(36)**,** 11929-11947. doi: 10.1523/JNEUROSCI.1860-14.2014.
